# Measuring the quality of skin cancer management in primary care: A scoping review

**DOI:** 10.1111/ajd.14023

**Published:** 2023-03-24

**Authors:** Samantha Spanos, Nehal Singh, Bela I. Laginha, Gaston Arnolda, David Wilkinson, Andrea L. Smith, Anne E. Cust, Jeffrey Braithwaite, Frances Rapport

**Affiliations:** ^1^ Centre for Healthcare Resilience and Implementation Science, Australian Institute of Health Innovation, Faculty of Medicine, Health and Human Sciences Macquarie University Sydney New South Wales Australia; ^2^ National Skin Cancer Centres South Brisbane Queensland Australia; ^3^ The Daffodil Centre University of Sydney, a joint venture with Cancer Council NSW Sydney New South Wales Australia; ^4^ Melanoma Institute Australia The University of Sydney Sydney New South Wales Australia

**Keywords:** general practitioners, primary care, quality, quality in healthcare, quality measurement, skin cancer, skin cancer management, variation

## Abstract

Skin cancer is a growing global problem and a significant health and economic burden. Despite the practical necessity for skin cancer to be managed in primary care settings, little is known about how quality of care is or should be measured in this setting. This scoping review aimed to capture the breadth and range of contemporary evidence related to the measurement of quality in skin cancer management in primary care settings. Six databases were searched for relevant texts reporting on quality measurement in primary care skin cancer management. Data from 46 texts published since 2011 were extracted, and quality measures were catalogued according to the three domains of the Donabedian model of healthcare quality (structure, process and outcome). Quality measures within each domain were inductively analysed into 13 key emergent groups. These represented what were deemed to be the most relevant components of skin cancer management as related to structure, process or outcomes measurement. Four groups related to the structural elements of care provision (e.g. diagnostic tools and equipment), five related to the process of care delivery (e.g. diagnostic processes) and four related to the outcomes of care (e.g. poor treatment outcomes). A broad range of quality measures have been documented, based predominantly on articles using retrospective cohort designs; systematic reviews and randomised controlled trials were limited.

## INTRODUCTION

Skin cancer is the most widespread form of cancer, with incidence rising worldwide.^1^, ^2^, ^3^ The most frequently diagnosed skin cancers are non‐melanoma skin cancers (NMSCs), mostly comprising the keratinocyte carcinomas (KCs), most of which are carcinomas of basal cells (BCCs) or squamous cells (SCCs).^4^ Melanoma is a rarer form of skin cancer, affecting melanocytic cells, representing 1.7% of all cancers in 2020.^5^ NMSC incidence is difficult to definitively determine because BCCs and SCCs are usually excluded from cancer registries.^6^, ^7^, ^8^

The highest incidence of both melanoma and NMSC is observed in predominantly fair‐skinned populations, such as those of Australia and New Zealand,^5^, ^9^ mostly due to high exposure to ultraviolet (UV) radiation from outdoor activities with insufficient sun protection.^10^ In Australia, for example, melanoma is the third most common major malignancy after prostate and breast cancer.^11^ NMSC is less likely to metastasise than melanoma,^12^ but as it has 18–20 times the incidence,^10^ NMSC and melanoma are both crucial parts of the skin cancer management challenge.^8^, ^13^, ^14^, ^15^

For common cancers, primary care practitioners typically focus on prevention and diagnosis, and support patients while coordinating with specialists.^16^ Many skin cancers, however, can potentially be managed entirely within the primary care setting^17^, ^18^, ^19^ and, as incidence increases, demand for GP consultations and treatment for skin lesions has also risen.^20^, ^21^

There has been a lack of formal recognition and definition of the roles and responsibilities of general practitioners (GPs) in treating and managing skin cancer.^22^, ^23^ Research has drawn attention to GPs' capabilities in managing skin cancer but also to concerns around variation in the quality of care.^22^, ^23^, ^24^, ^25^ High levels of variability in diagnostic accuracy have been found between individual GPs,^26^, ^27^ and high variability in GPs familiarity with best practice guidance on high‐risk excisions^28^ and use of sentinel lymph node biopsy.^29^

Skin cancer focused protocols and guidelines have been developed by dermatological and oncological societies (e.g. for surgical excision^30^), but these have rarely detailed the role to be played by primary care.^31^, ^32^, ^33^, ^34^ GPs' approaches to skin cancer care have been found to be most influenced by their own training, interests, expertise and interactions with patients and colleagues.^35^, ^36^, ^37^, ^38^

Development of guidelines is insufficient to ensure high‐quality care. Implementation of quality indicators, measurable elements of practice performance derived from guidelines, allow primary care practitioners to benchmark their performance against peers.^39^, ^40^, ^41^, ^42^, ^43^ The Donabedian model of healthcare quality proposes that measures can relate to structure (i.e. attributes of settings), process (i.e. the giving and receiving of care) or outcome (i.e. effects of care on health status), with good structure and process contributing to better outcomes.^44^, ^45^

A set of quality indicators for the diagnosis and management of early stage cutaneous melanoma was recently developed,^46^ targeting readily available measures of care processes such as pathology results.^46^ It is also important to address the influence of setting (i.e. primary care) on the utility of quality indicators.^47^ For example, is there a system in place to allow data to be understood and acted upon? Barriers to implementing quality measures differ across settings^42^, ^48^, ^49^ and thus structural measures can affect clinicians' approaches to local quality improvement.

The aim of this scoping review was to better understand the literature on quality measurement of skin cancer management in primary care settings over the past decade.^44^ Our approach was to keep the review broad, not limited to specific quality indicators that have been formally implemented or standardised, in order to understand the range and breadth of possible skin cancer care quality measures. Specific research questions relating to primary care skin cancer management were:

1. What types of evidence informs the measurement of quality?
2. What key groups of quality measurement have been explored or proposed?

## MATERIALS AND METHODS

Relevant details relating to this study, and the project of which it is part, have been described elsewhere.^50^ Selected details are described below.

### Search strategy

A detailed search strategy was developed in association with an electronic information search expert (medical librarian) to optimise within each database the identification of relevant articles.^51^, ^52^ Six databases were searched on 1 December, 2021: Medline, PsycINFO, Embase, Scopus, CINAHL and Cochrane Library (see Appendix S1 for Medline search strategy). Searches were conducted in accordance with the Preferred Reporting Items for Systematic Reviews and Meta‐Analyses Extension for Scoping Reviews (PRISMA‐ScR) guidelines.^53^ Where a selected article identified another article that contained relevant information, and the other article was also found within our initial six‐database search but excluded during screening, that article was also included in the review. This restricted snowballing was used to protect against the inadvertent exclusion of relevant articles during screening.

### Article selection

References were extracted into Endnote and duplicates identified and removed. References were uploaded into Covidence where titles and abstracts were screened by two team members (BIL screened all references, and the second reviewer was either LvB, DW, AEC, AS, CL, KH, MB, or FR) to assess compliance with inclusion and exclusion criteria (Table 1). If reviewers disagreed, a third reviewer (NS or LvB) facilitated consensus

**TABLE 1:** Inclusion and exclusion criteria for study selection in scoping review.

Inclusion criteria:
Articles reporting on skin cancer/skin lesions/neoplasms (benign or malignant), non‐melanocytic skin cancers and/or pre‐cancerous skin lesions
Articles reporting in the context of primary care; reference was made to the primary care consultation itself or any related follow‐up/monitoring phase
Articles reporting on specific quality indicators or the use of performance outcomes as a measure of quality
Exclusion criteria:
Articles reporting on skin cancer management exclusively in secondary or tertiary care
Articles reporting on training programs for resident/training doctors
Articles reporting on performance outside of clinical practice (e.g. testing diagnostic accuracy on images)
Articles focused on the effectiveness of diagnostic tools based on dermatologist diagnosis
Editorials, commentaries and letters
No full‐text available
Protocols
Articles published prior to 2011, to focus on contemporary practice

Full‐text reviews were conducted by five team members (SS, NS, DW, BIL and LvB). Each article was independently read in full by two team members and assessed for eligibility. Disagreements were resolved through discussion; if needed, a third reviewer was consulted.

### Data charting process

Data were extracted into a Microsoft Excel spreadsheet by two authors (SS and NS) and independently checked for accuracy (SS or GA). Extracted data were categorised as article details (authors, year, country, text type, objectives, conclusions, implications), study details (article type/study design, data source, setting, primary vs. secondary data, intervention type, control/comparison type), sample characteristics (type, size, attrition, gender, age, lesion type) and outcomes (type of quality measure, data source, indicator numerator and denominator). Where applicable, information about implementation was also extracted (acceptability, feasibility, reliability, validity).

### Synthesis of results

Data from included articles were analysed by describing the breadth, range and type of included data and thematic analysis^54^, ^55^, ^56^ to identify the underlying groups of quality proposed for measurement. Two team members (SS and NS) categorised measures according to the structure, process, outcome domains,^44^ extracting data on a master sheet. SS and NS reviewed quality measures within each domain, discussed and generated a set of codes to represent the data, and summarised these codes into groups and subgroups of quality measurement. SS and NS met regularly to discuss discrepancies and reach consensus on categorisation and synthesis, consulting with GA regularly. Consensus‐building teamwork during qualitative analysis helped confirm the trustworthiness of data and the veracity of resulting groups and subgroups.^57^

## RESULTS

### Search results

**FIGURE 1:**
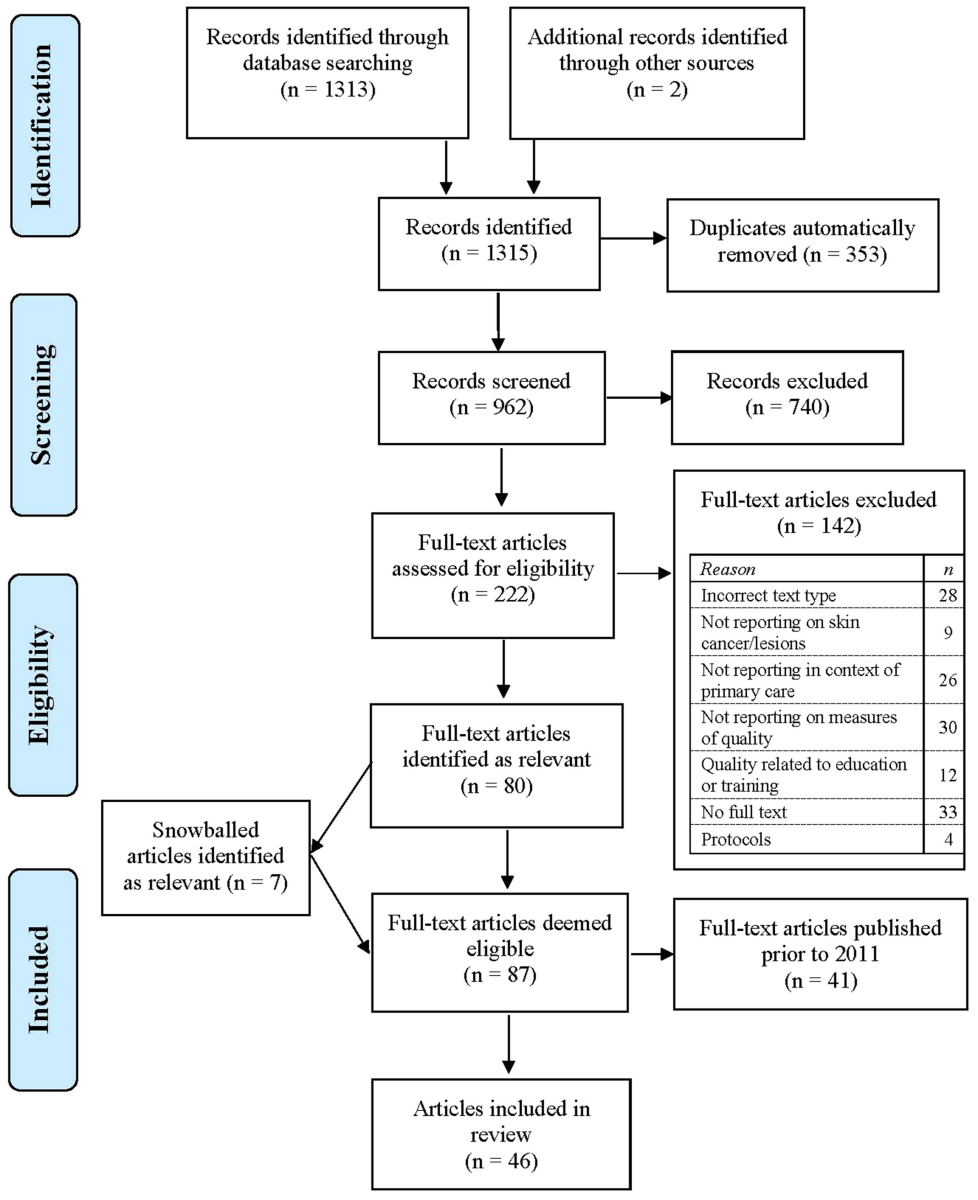
PRISMA flowchart displaying the process of identification and selection of included articles.

As shown in Figure 1, 1315 references were identified, of which 353 were duplicates, leaving 962 articles for title and abstract screening. Of these, 740 did not meet eligibility criteria, leaving 222 articles for full‐text review. After full‐text review, 142 failed to meet the eligibility criteria (see reasons in Figure 1) leaving 80 articles. An additional seven articles were identified through snowballing. After removing 41 articles published before 2011, 46 articles were retained for review.

### Characteristics of reviewed articles

The characteristics of included articles are displayed in Tables 2 and 3. Twenty articles (43%) were published from 2011 to 2016 and 26 (57%) were published from 2017 to 2022. Most articles were conducted and/or published in Europe (*n* = 29; 63%), particularly in the United Kingdom (*n* = 12; 41% of European articles). The rest came from North America (20%) and Australasia (17%). Six articles were practice guidelines or recommendations (13%), five were systematic reviews (11%), one was a clinical literature review and one used a modified Delphi approach, with the remaining 33 having the following designs: retrospective cohort^21^; cross‐sectional,^7^ two of which also had cohort elements; prospective cohort^4^; and randomised controlled trial (RCT; 3).

**TABLE 2:** Selected characteristics of articles included in scoping review.

Study	Country of study	Article type or study design	Skin lesion/cancer type	Data source
Ahmadi (2017)^17^	The Netherlands	Retrospective cohort	Lesions suspected of malignancy	Medical records
Aung (2019)^95^	Australia	Prospective cross‐sectional	BCC, SCC, melanoma	Questionnaires and interviews
Bibbins‐Domingo (2016)^66^	USA	Recommendations	N/A	Systematic review data
Blood (2021)^96^	Australia	Systematic review	Melanoma	Clinical quality registries
Botting (2016)^71^	UK	Prospective cohort	Lesions surgically removed	Clinical data collected
Buckley (2013)^79^	UK	Retrospective cohort	Melanoma	Medical records
Chuh (2020)^58^	Switzerland	Recommendations	N/A	N/A
Cole (2018)^28^	UK	Retrospective cohort	BCC	Medical records
Delaney (2012)^23^	UK	Retrospective cohort	SCC	Medical records
Dinnes (2018a)^60^	UK	Systematic review	BCC, SCC	Prior studies (data on dermoscopy vs. visual inspection)
Dinnes (2018b)^59^	UK	Systematic review	Melanoma	Prior studies (data on dermoscopy vs. visual inspection)
Doherty (2016)^80^	Ireland	Retrospective cohort	Melanoma	Cancer registry
Gendreau (2017)^77^	USA	Retrospective cohort	Melanoma	Medical records, veteran registry, teledermatology registry
Guitera (2021)^78^	Australia	Prospective cohort	Melanoma	Clinical data collected, medical records
Hajdarevic (2014)^81^	Sweden	Retrospective cohort	Melanoma	Melanoma registry, medical records
Haw (2014)^82^	UK	Retrospective cohort	BCC, SCC, melanoma	Medical records
Hay (2022)^83^	Australia & New Zealand	Retrospective cross‐sectional	Melanoma	Skin Cancer Audit Research Database (SCARD)
Heppt (2020)^69^	Germany	Guideline	Actinic keratosis, SCC	Expert consensus
Herschorn (2012)^61^	Canada	Clinical literature review	Melanoma	Prior studies (data on dermoscopy vs. visual inspection)
Jimenez Balcells (2021)^97^	Australia & New Zealand	Retrospective cross‐sectional	Melanoma	Skin Cancer Audit Research Database (SCARD)
Jobson (2022)^46^	Australia	Modified Delphi	Melanoma	Expert consensus
Kaiser (2014)^84^	USA	Retrospective cohort	Melanoma	Cancer registry
Koelink (2014)^62^	The Netherlands	Randomised controlled trial	Lesions suspected of malignancy	Clinical data collected, medical records
Korgul (2018)^37^	UK	Retrospective cohort, cross‐sectional questionnaire	BCC, SCC, melanoma	Medical records
Leiter (2020)^73^	Germany	Guideline	Actinic keratosis, SCC	Expert consensus
Lott (2015)^89^	USA	Retrospective cohort	Melanoma	Health insurance registry
Maguire (2017)^92^	Ireland	Retrospective cohort	BCC, SCC	Medical records
Martinka (2016)^75^	Canada	Retrospective cohort	Melanoma	Medical records
Moreno‐Ramirez (2016)^70^	Spain	Guideline	Benign skin tumours	Expert working group
Moyer (2012)^74^	USA	Recommendations	N/A	Literature review data
Murchie (2013)^86^	UK	Retrospective cohort	Melanoma	Melanoma registry, death registry, medical records
Murchie (2017)^85^	UK	Retrospective cohort	Melanoma	Cancer registry, death registry, medical records
Noels (2019)^67^	The Netherlands	Retrospective cohort	Actinic keratosis	Cohort study data, medical records, health claims data
Nolan (2021)^94^	UK	Systematic review	BCC, SCC	Prior studies (data on excision completeness)
Ramdas (2018)^93^	The Netherlands	Retrospective cross‐sectional	BCC	Medical records
Renzi (2011)^90^	Italy	Retrospective cohort and cross‐sectional interviews	SCC	Medical records, interviews
Smith (2014)^72^	Australia	Randomised controlled trial	Lesions surgically removed	Clinical data collected
Svensson (2020)^87^	Sweden	Retrospective cohort	SCC	Medical records
Van Rijsingen (2015)^76^	The Netherlands	Retrospective cohort	Lesions surgically removed	Medical records
Vestergaard (2020)^63^	Denmark	Prospective cohort	Lesions suspected of malignancy	Clinical data collected, questionnaire
Wakkee (2019)^34^	The Netherlands	Retrospective cohort	BCC, SCC	Cancer registry, medical records
Walter (2012)^64^	UK	Randomised controlled trial	Lesions suspected of malignancy	Clinical data collected, questionnaires
Wen (2020)^91^	New Zealand	Retrospective cohort	Lesions surgically removed	Medical records
Wernli (2016)^68^	USA	Systematic review	N/A	Prior studies (data on skin cancer screening)
Wheatley (2018)^65^	USA	Prospective cohort	N/A	Quality improvement methodology
Wikstrom (2018)^88^	Sweden	Retrospective cross‐sectional	Melanoma	Medical records, interviews

Abbreviations: BCC, basal cell carcinoma; SCC, squamous cell carcinoma.

**TABLE 3:** Frequency of study characteristics included in scoping review.

Study characteristics (*N* = 46)	*n* (%)
Publication year	
2011–2013	7 (15)
2014–2016	13 (28)
2017–2019	14 (30)
2020–2022	12 (26)
Study location	
Europe	29 (63)
North America	9 (20)
Australasia	8 (17)
Article type or study design^a^	
Guidelines and recommendations	6 (13)
Modified Delphi	1 (2)
Systematic review	5 (11)
Clinical literature review	1 (2)
Randomised controlled trial	3 (7)
Prospective cohort	4 (9)
Retrospective cohort	21 (46)
Cross‐sectional	7 (15)
Location of data collection^b^	
Urban	26 (57)
Regional	10 (22)
Rural	3 (7)
Not reported or not applicable	11 (24)
Skin lesion/cancer type examined^c^	
Melanoma	20 (43)
Squamous cell carcinoma	12 (26)
Basal cell carcinoma	9 (20)
Actinic keratosis	3 (7)
Benign skin lesions	1 (2)
Non‐specific lesion types	8 (17)
Type of quality measures examined^d^	
Structure	18 (39)
Process	44 (96)
Outcome	17 (37)

Abbreviations: *N*, total number of articles included in scoping review; *n*, number of articles included in the frequency analysis.

^a^ Two articles included both cohort and cross‐sectional designs.

^b^ Several articles took place in more than one location.

^c^ For which the skin lesion/cancer was a specific focus of the study.

^d^ Articles often contained more than one type of quality measure.

### Groups of quality measurement

Thirteen groups of quality measurement emerged through thematic analysis (see Table S1 for the authors that contributed to each group).

### Structure measures of quality

Eighteen articles (39%) evaluated or proposed potential quality measures relating to structural elements of care provision; four groups of quality measures were derived (Table 4).

**TABLE 4:** Quality measures relating to structures of skin cancer care.

Group	Subgroup	Examples	*n* (%)
Diagnostic tools and equipment	Inspection aids and imaging systems^58^, ^59^, ^60^, ^61^, ^62^, ^63^, ^64^, ^78^	Establish a platform for storing and retrieving clinical and dermoscopic images^58^	8 (17)
		Impact of dermoscope on detection of melanoma compared to visual examination^61^	
Practitioner education and training	Visual skin inspections^65^	Education on the importance of proper skin inspections and appropriate documentation of abnormal skin findings^65^	6 (13)
	Diagnostic tool‐assisted skin inspections^59^, ^60^, ^61^, ^62^, ^64^	Dermoscopy course focused on distinguishing between melanocytic and non‐melanocytic lesions^62^	
Diagnostic protocols and documentation	Community screening^65^, ^66^, ^67^, ^68^	Full‐body skin examination (FBSE) for community members by dermatologically trained physicians^67^	13 (28)
	Diagnosing suspect lesions^58^, ^59^, ^60^, ^61^, ^62^, ^63^, ^64^, ^69^, ^70^	Use of checklists for meeting dermoscopy standards of use for patients with suspected basal cell carcinoma diagnosis^60^	
Treatment protocols and documentation	Surgical and procedural safety^58^, ^69^, ^70^, ^71^, ^72^, ^73^	Surgical wound management protocol for standardised excision management^72^	6 (13)
		Surgery audit form filled out by practitioners completing minor surgeries^71^	

Abbreviations: *n*, number of articles included in the thematic analysis.

#### Diagnostic tools and equipment

Eight articles evaluated the effectiveness of diagnostic tools and equipment, falling within a single subgroup of *inspection aids and imaging systems*. These articles focused primarily on the use of dermoscopy and other diagnostic aids (e.g. MoleMate system), but also addressed image storage and retrieval platforms.^58^, ^59^, ^60^, ^61^, ^62^, ^63^

#### Practitioner education and training

Six articles evaluated the impact of education and training programs on clinical practice. Most of these articles examined the effect of education and training for *diagnostic tool‐assisted skin inspections* on detection accuracy,^59^, ^60^, ^61^, ^62^, ^64^ while one sought to improve *visual skin inspection*.^65^

#### Diagnostic protocols and documentation

Thirteen articles assessed protocols and procedures to facilitate *community or routine screening* ^65^, ^66^, ^67^, ^68^ or for the purpose of *diagnosing suspicious lesions*.^58^, ^59^, ^60^, ^61^, ^62^, ^63^, ^64^, ^69^, ^70^ These articles recommended dermoscopy checklists and algorithms,^58^, ^59^, ^60^, ^61^ standardised recording forms^65^, ^66^, ^67^ and visual skin examination checklists.^66^

#### Treatment protocols and documentation

Six articles^58^, ^69^, ^70^, ^71^, ^72^, ^73^ presented protocols and procedures for treatment, within a single subgroup of *surgical and procedural safety*. Recommendations included the use of guidelines for surgical safety,^58^, ^69^, ^70^, ^73^ surgical audit forms^71^ and antibiotics use to prevent infection.^72^

### Process measures of quality

Forty‐four articles (96%) evaluated or proposed potential quality measures relating to care provision, across five groups (Table 5).

**TABLE 5:** Quality measures relating to processes of skin cancer care.

Group	Subgroup	Examples	*n* (%)
Prevention	Early prevention^74^	Primary care‐based counselling on ultraviolet exposure reduction for people aged 10–24 years with fair skin^74^	3 (7)
	High‐risk surveillance^69^, ^73^	Using the preventive effects of ultraviolet (UV) radiation protection and vitamin B6 on AK progression^69^	
		Information on the hazards of occupational UV radiation and behaviour change recommendations for workers with occupational exposure to UV radiation^73^	
Diagnostic process	Unassisted visual diagnosis^17^, ^66^, ^68^, ^69^, ^70^, ^71^, ^75^, ^76^	Proportion of correct diagnoses of melanoma by physician (compared to dermatologist diagnosis as gold standard)^75^	29 (63)
		Sensitivity and specificity for melanoma detection by dermatologists and GPs during clinical skin cancer screening^68^	
	Diagnostic‐tool assisted diagnosis^17^, ^59^, ^60^, ^61^, ^62^, ^63^, ^64^, ^69^, ^70^, ^77^, ^78^	Proportion of melanomas that were found with the aid of total‐body photography or sequential digital dermoscopy imaging^78^	
		Odds ratio of correctly diagnosed lesions with a dermoscope versus without a dermoscope^62^	
	Diagnostic biopsy performance^17^, ^23^, ^34^, ^46^, ^62^, ^69^, ^71^, ^75^, ^76^, ^79^, ^80^, ^81^, ^82^, ^83^, ^84^, ^85^, ^86^, ^87^	Proportion of positive cutaneous squamous cell carcinoma biopsies that were punch biopsies^23^	
		Proportion of excisional biopsies on melanoma and non‐melanoma skin cancer^76^	
	Patient staging^13^, ^46^, ^69^, ^73^	Proportion of primary invasive melanomas for which sentinel lymph node biopsy was discussed^13^	
		Radiological scans should not be performed on asymptomatic patients with stage 0–II disease^46^	
Delays in care	Biopsy delays^77^, ^79^, ^81^, ^88^	Average time taken by GP from first consultation to biopsy in patients with suspected melanoma^79^	8 (17)
	Pathology delays^71^, ^81^, ^88^	Time interval (delays) from primary excision until registration of histopathological diagnosis in patients with melanoma^81^	
	Referral delays^81^, ^88^	Referral lead time between primary care and university level care^88^	
	Treatment delays^71^, ^81^, ^88^, ^89^, ^90^, ^91^	Surgical delay of 1.5 months from biopsy to excision in patients with melanoma^89^	
Treatment process	Excision performance and adequacy^17^, ^23^, ^25^, ^28^, ^34^, ^37^, ^46^, ^62^, ^66^, ^68^, ^69^, ^71^, ^72^, ^73^, ^76^, ^78^, ^79^, ^80^, ^81^, ^82^, ^83^, ^85^, ^87^, ^88^, ^91^, ^92^, ^93^, ^94^	Proportion of excisions performed on skin lesions suspected of malignancy^17^	32 (70)
		Rate of incomplete excisions of non‐melanoma skin lesions^92^	
	Other surgical treatment^46^, ^66^, ^69^, ^71^, ^73^, ^87^	Proportion of squamous cell carcinomas treated by curettage^87^	
		Completion lymph node dissection should not be performed following a positive sentinel lymph node biopsy^46^	
	Non‐surgical treatment^17^, ^34^, ^67^, ^69^, ^72^, ^73^, ^79^, ^92^	Proportion of non‐melanoma skin lesions treated using cryotherapy^92^	
		Proportion of melanomas treated with imiquimod^79^	
	Post‐treatment follow‐up^34^, ^67^, ^70^, ^72^, ^73^, ^78^, ^81^, ^88^, ^95^	Proportion of melanoma patients requiring follow up after initial excision in primary care^81^	
		Use of patient recall systems for each skin cancer type^95^	
Interpersonal process	Communication with patient^58^, ^88^	Proportion of melanoma diagnoses communicated in‐person, via phone and via post^88^	4 (9)
	Assessing patient care experience^64^, ^88^, ^96^	Proportion of patients reporting satisfaction with melanoma care at post‐surgery follow‐up^88^	
		Proportion of patient satisfaction surveys completed after lesion assessment within 1 week of consultation^64^	

Abbreviations: *n*, number of articles included in the thematic analysis; GP, general practitioner.

#### Prevention

Three articles identified measures related to prevention. Behavioural counselling for younger patients was recommended as *early prevention* by US Preventative Services Task Force^74^ and re‐iterated.^66^ Two guideline articles recommended *high‐risk surveillance* practices including monitoring skin damage, UV light exposure and occupational risk factors.^69^, ^73^

#### Diagnostic processes

Twenty‐nine articles identified measures relevant to diagnosis‐related processes of care, in four subgroups. These articles evaluated diagnostic accuracy relative to a gold standard (e.g. histopathology diagnosis or comparison to dermatologist) either as *unassisted visual diagnosis* ^17^, ^71^, ^75^, ^76^ or as *diagnostic tool‐assisted diagnosis*.^59^, ^60^, ^61^, ^62^, ^63^, ^64^, ^77^, ^78^ Eighteen articles evaluated *diagnostic biopsy performance*,^17^, ^23^, ^34^, ^46^, ^62^, ^69^, ^71^, ^75^, ^76^, ^79^, ^80^, ^81^, ^82^, ^83^, ^84^, ^85^, ^86^, ^87^ including the proportion of biopsy types performed (e.g. excision biopsies^34^ vs. shave or punch biopsies^84^), and biopsy performance comparisons between primary care practitioners and other skin specialists.^75^, ^82^ Three articles focused on treatment workup and *patient staging* for more complex cases^46^, ^69^, ^73^, ^83^. Two articles were guidelines to achieve optimum diagnostic accuracy, with and without diagnostic tools, and enhance biopsy performance.^69^, ^70^

#### Delays in care

Eight articles assessed delays in care. Delays were defined in terms of the time between: GP consultation and biopsy (*biopsy delay*)^77^, ^79^, ^81^, ^88^; biopsy submitted and diagnosis received or communicated to patients (*pathology delay*)^71^, ^81^, ^88^; results received and referral (*referral delay*)^81^, ^88^; and results received and treatment (*treatment delay*).^71^, ^81^, ^88^, ^89^, ^90^, ^91^

#### Treatment processes

Thirty‐two articles examined treatment processes of care. Evaluations of *excision performance and adequacy* of GPs (88%) usually measured the proportion of skin cancers excised^17^, ^28^, ^34^, ^62^, ^76^, ^78^, ^79^, ^80^, ^81^, ^82^, ^83^, ^85^, ^86^, ^87^, ^88^, ^91^, ^92^, ^93^ or the proportion of complete (vs. partial) excisions.^23^, ^28^, ^34^, ^37^, ^71^, ^76^, ^82^, ^83^, ^86^, ^87^, ^91^, ^92^, ^93^, ^94^ *Other surgical treatment procedures*, such as curettage, were also examined,^46^, ^66^, ^69^, ^71^, ^73^, ^87^ as well as *non‐surgical treatment* such as cryotherapy.^17^, ^34^, ^67^, ^69^, ^72^, ^73^, ^79^, ^92^ *Post‐treatment follow‐up* proposed different follow‐up practices and systems^78^, ^88^, ^95^ and assessed follow‐up visit completion rates.^34^, ^67^, ^81^ Two articles provided consensus‐based recommendations for patients with skin lesions.^70^, ^73^

#### Interpersonal process

Four articles examined the interpersonal aspects of care.^44^ *Communication with patients* assessed methods of communication.^58^, ^88^ Four articles focused on *assessing patient experience* by measuring the proportion of patient‐reported measures (PRMs) completed,^64^, ^88^ and the collection rates of PRMs for clinical registries.^96^

### Outcome measures of quality

Seventeen articles (37%) evaluated or proposed quality measures relating to outcomes of care, in four groups (Table 6).

**TABLE 6:** Quality measures relating to outcomes of skin cancer care.

Group	Subgroup	Examples	*n* (%)
Treatment complications and adverse events	Post‐operative infections^71^, ^72^, ^91^	Proportion of surgeries for which infection occurred within 2 months^71^	6 (13)
		Rate of wound infections in patients with lower limb excisions^72^	
	Short‐term morbidity^67^, ^85^, ^86^	Total number of inpatient and outpatient attendances from the date of melanoma diagnosis^86^	
		Treatments, follow‐up visits and potential subsequent claims for cutaneous malignancies in patients previously diagnosed with actinic keratosis^67^	
Patient reported measures	Patient satisfaction with care^64^, ^88^, ^96^	Patient satisfaction with care received by a GP, private consultant and in a university hospital^88^	3 (7)
		Patient satisfaction survey related to quality of melanoma care provided by GPs^88^	
	Patient‐reported health outcomes^64^, ^96^	Registries specific for melanoma favoured the use of health‐related quality of life (HR‐QoL) PROMs^96^	
		Patients' anxiety measured by questionnaire completed within 1 week and at 3 months after clinician consultation^64^	
Post‐treatment skin cancer recurrence	Non‐melanoma recurrence rates^34^, ^67^, ^92^	Proportion of patients with non‐melanoma skin lesions excised that had a non‐melanoma skin lesion reoccur^92^	6 (13)
		Frequency of documented basal cell carcinoma and squamous cell carcinoma during follow‐up of patients with suspected actinic keratosis^67^	
	Melanoma recurrence rates^78^, ^79^, ^83^	Proportion of melanomas excised for which a subsequent lesion arose^83^	
		Proportion of treated melanoma patients for which lesions recurred^79^	
Long‐term morbidity and mortality	Morbidity^68^, ^79^, ^83^, ^97^	Association between earlier detection of skin cancer and skin cancer morbidity^68^	7 (15)
		Proportion of patients with invasive melanoma that progressed to metastatic disease^83^	
	Mortality^68^, ^80^, ^83^, ^85^, ^86^, ^97^	Mortality rate for melanoma patients who had lesions excised in primary care^85^	
		Associations between tumour thickness and skin cancer mortality^68^	

Abbreviations: *n*, number of articles included in the thematic analysis; GP, general practitioner; PROMs, patient‐reported outcome measures.

#### Treatment complications and adverse events

Six articles assessed treatment complications and adverse events such as *post‐operative infections*,^35^, ^71^, ^91^ as well as *short‐term morbidity* indicated by post‐treatment hospital admissions^85^, ^86^ and subsequent treatments.^67^

#### Patient‐reported measures

Three articles evaluated PRMs, focused on *patient satisfaction with care* provided as cancer treatment^64^, ^88^ or *patient‐reported health outcomes* such as anxiety or condition improvement.^64^ One article reviewed implementation of patient‐reported experience measures in practice.^96^

#### Post‐treatment recurrence of skin cancer

Six articles examined skin cancer recurrence rates, including *NMSC recurrence* after lesion excision^92^ or suspected AK,^67^ and *melanoma recurrence* post‐melanoma surgery^78^, ^79^ or post‐AK diagnosis.^67^

#### Long‐term morbidity and mortality

Seven articles assessed long‐term morbidity and mortality. *Morbidity* was measured as the proportion of cases that progressed to metastasis,^79^, ^83^, ^97^ including from time of detection.^68^ *Mortality* was measured as the proportion of cases that resulted in skin cancer death^80^, ^83^, ^85^, ^97^ or as a function of tumour thickness.^68^, ^97^

## DISCUSSION

### Types of articles

This scoping review identified 46 articles that suggest possible quality measures relevant to primary care skin cancer management, over the last decade. Most assessed skin cancer care quality through retrospective cohort articles, a design that provides valuable insights when RCTs are not feasible,^98^ and a commonly employed to assess care quality.^99^, ^100^ Three RCTs assessed elements of care quality.^62^, ^64^, ^72^ Five systematic reviews were identified, three with a meta‐analytic component.^59^, ^60^, ^94^

### Quality measurement

Thirteen groups of activities that may be suitable for quality measurement were derived. Most widely considered over the last decade are process measures, often referred to as ‘intermediate outcomes’ that provide actionable data on clinical and management processes in a timely manner, and thus are the most frequently utilised quality measures.^41^, ^101^, ^102^ Five groups of process measures were identified: prevention, diagnostic process, delays in care, treatment process and interpersonal process.

Diagnostic accuracy, a common focus, was assessed predominantly by comparing GPs diagnosis (either visually or tool‐assisted) with histopathological^71^ or dermatologist diagnosis.^75^ The proportion of partial versus full excision biopsies has been proposed of a measure of care quality, but its usefulness has been questioned, suggesting the need for further development.^103^, ^104^

Delays in care were assessed by examining lead times between initial contact to diagnosis and treatment, to identify where care can be improved, particularly for patients with more advanced skin cancer.^81^ Caution is needed, however, as lead times may also reflect the time needed to engage family in treatment planning, and to manage complex patients, factors which must be controlled for when comparing delays in care.^81^, ^89^

Surgical performance was the common focus of treatment process quality, often assessed from histopathology reports, to calculate the proportion of lesions excised,^76^ and the proportion of excisions that were complete.^23^ Some concerns with excision performance as a measure of quality relate to inaccuracies in GP recording of histopathological clearance,^92^ whether ‘near to’ excised lesions were considered complete,^94^ selection bias in the subset of patient data examined^17^, ^94^ and lack of longer‐term follow‐up of recurrence rates to definitively establish surgical quality.^92^

Many articles assessing diagnostic and treatment quality used medical records as their primary data source. Medical records depend heavily on sound documentation—which is often lacking.^42^, ^48^, ^67^ Incomplete records could potentially lead to underestimating GPs diagnostic accuracy,^17^, ^67^ or fail to document patient risk factors contributing to excision,^82^ or misrepresent surgical adequacy,^92^, ^94^ or inaccurately depict follow‐up care.^67^, ^88^ Inaccurate or incomplete documentation, and lack of standardisation in histopathological data collection and analysis systems, are major barriers to the reliability of audit and feedback.^105^, ^106^, ^107^

Relatively few articles assessed interpersonal aspects of care. Two discussed patient‐centred communication during care delivery,^58^, ^88^ while patient experience post‐care was assessed in two articles through patient questionnaires.^64^, ^88^ Increasing commitment to patient‐centred care suggests that facilitating shared decision‐making could be explored in skin cancer care.^108^, ^109^

Structural measures of quality from the included articles related to *diagnostic tools and equipment*, *practitioner education and training*, and *protocols and documentation systems* (separately for diagnosis and treatment). Two of the three RCTs included in this review addressed the effectiveness of skin inspection aids and imaging systems on diagnostic accuracy.^62^, ^64^ Two articles investigated the feasibility of implementing diagnostic aids into practice,^62^, ^63^ and two looked at barriers to implementation.^61^, ^78^ A common challenge cited was that tools are usually evaluated in specialist settings rather than primary care populations^62^, ^78^, ^84^ which have lower incidence on presentation and lower patient volumes.

Documentation systems across diagnosis and treatment included visual examination checklists,^64^ dermoscopy algorithms^58^ and case report forms.^63^ Education and training programs were often assessed as part of interventions to improve clinical practice^62^, ^64^, ^65^ or in reviews evaluating diagnostic accuracy.^59^, ^60^, ^61^ Structural measures, on their own, provide limited inferences about care quality,^110^ but often relate to minimum or ideal standards.

Outcome measures were also identified in the reviewed articles, including externally recorded outcomes and *patient‐reported measures*. Externally recorded outcomes included post‐*treatment complications and adverse events* (e.g. hospital admissions^67^), *post‐treatment skin cancer recurrence*,^83^ and *longer‐term morbidity* (e.g. rate of metastasis^97^) *and mortality*.^68^ Although outcome measures can be used to detect trends and identify outliers,^102^ their validity and reliability as quality indicators is contentious due to the multitude of patient‐ and measurement‐related confounders.^44^, ^110^, ^111^, ^112^ Evaluation of commoner outcomes can be improved by controlling for population risk and other covariates^113^, ^114^; rarer outcomes like mortality, however, are acknowledged as insensitive measures of care quality even after adjustment except at the macro level.^115^

Patient‐reported outcome and experience measures are increasingly a focus of quality measurement,^116^ collected prospectively in two included articles.^64^, ^88^ Patient perceptions of skin cancer treatment outcomes can substantially influence their health and quality of life,^117^ but PRMs are challenging to implement in routine practice due to time and cost constraints,^96^ limiting their routine deployment.

Data sources used to assess care quality must be valid and reliable, considered appropriate by clinicians and patients, and feasible to implement in practice.^40^, ^110^, ^118^ Structure, process and outcomes of care are inherently linked, so the relationships between them must be understood for a comprehensive assessment of healthcare quality in different settings.^44^, ^45^, ^111^ Ideally, RCTs could provide evidence that compliance with specific structure and process quality measures leads to improvements in specific outcomes.^45^, ^110^

### Strengths and limitations

This scoping review cast a wide net to capture the ways in which quality has been conceptualised in primary care skin cancer management over the last decade. The thematic framework identified presents broad groupings of the structure, process and outcome quality measures proposed in primary care skin cancer management and can help to inform the development of primary care guidelines, from which indicators can be derived.

This review has several limitations. Although the search strategy was designed to comprehensively capture a broad scope of quality measurement, the search terms selected may not have adequately captured literature related to key issues such as the administrative structures and organisation of services that contribute to care quality.^111^ In radiation therapy for cancer, for example, facilities are regularly surveyed, within and across nations, to inform guidance on minimum or ideal resource levels.^119^

In addition, restricting our database searches to articles indexed with keywords related to ‘quality indicators’ may have led to the exclusion of important articles on primary care skin cancer management. For example, a reviewer brought to our attention an important article^27^ that addresses dermoscopy use, which was not identified through our searches or through snowballing and did not meet our inclusion criteria. It is important to note that the authors reviewed the ineligible article and concluded that had it been included it would not have altered the groupings we derived from thematic analysis of the included papers. While the weaknesses of the search strategy may detract from the richness of the data, this example suggests that the groupings derived from the included articles are robust.

As a separate limitation, we aimed to capture important quality measures suggested or proposed by each article, but it is beyond our scope to analyse in detail each individual finding as a potential indicator. It was also beyond our scope to attempt to draw conclusions about the groups or subgroups that are of greatest priority; feasibility of measurement is important to identifying indicators suitable for early adoption, but ultimately a comprehensive coverage of all the dimensions of quality is desirable. A comprehensive item‐specific evidence review will be required to inform a guideline development process.

## CONCLUSIONS

This scoping review has identified 13 groups of structure, process and outcome measures that have been suggested or proposed to assess quality in skin cancer management in primary care settings. This review highlights the range of areas in which relevant indicators need to be considered for development.

## FUNDING INFORMATION

This work was in part funded by the National Skin Cancer Centres (NSCC). This work is also supported by the Australian Institute of Health and Innovation (AIHI) at Macquarie University, the National Health and Medical Research Council (NHMRC) Centre of Research Excellence in Melanoma (CRE grant number: 1135285) and the NHMRC‐funded Centre of Research Excellence in Implementation Science in Oncology (CRE grant number: 1135048). AEC is funded and supported by an NHMRC Investigator Grant (2008454). JB is funded and supported by an NHMRC Leadership Investigator Award (1176620).

## CONFLICT OF INTEREST STATEMENT

DW is a member of the National Skin Cancer Centres board of directors.

## Supporting information


Appendix S1.

Table S1.

## Data Availability

Data supporting these research findings are available in the supplementary material and further inquiries can be directed to the corresponding author.
