# Supplementary material for: Measuring the quality of skin cancer management in primary care: A scoping review
Source: Australas J Dermatol. 2023 Mar 24;64(2):177–93. doi: 10.1111/ajd.14023 (PMC10952799; doi:10.1111/ajd.14023)
Supplement: Supplementary file 1 — Appendix S1. [file AJD-64-177-s002.docx]

**Appendix S1: Medline Search Strategy**

The search strategy for Medline adapted to create search strategies for the remaining five databases (PsycINFO, Embase, Scopus, CINAHL, Cochrane Library), for which search terms were adjusted slightly based on differences in database subject headings.

| **Criteria** | **Search terms** |
| --- | --- |
| [skin cancer] | 1. skin neoplasms/ or acanthoma/ or skin cancer*.tw. |
|  | 1. carcinoma, basal cell/ or carcinoma, squamous cell/ or bowen's disease/ |
|  | 1. melanoma/ or hutchinson's melanotic freckle/ or melanoma, amelanotic/ |
|  | 1. 1 or 2 or 3 |
| [primary care] | 1. Primary Health Care/ |
|  | 1. general practice/ or family practice/ or general practitioners/ |
|  | 1. (general practi* or gp or family practice or family doctor or (primary adj2 health*) or (skin adj1 cancer adj1 clinic)).tw. |
|  | 1. 5 or 6 or 7 |
| [quality indicators] | 1. Quality Improvement/ or Quality control/ |
|  | 1. "outcome and process assessment, health care"/ or exp outcome assessment, health care/ or process assessment, health care/ or quality assurance, health care/ or benchmarking/ or "Quality of Health Care"/ or Health plan implementation/ |
|  | 1. quality indicators, health care/ or risk adjustment/ or "standard of care"/ or (quality adj1 indicator*).tw. |
|  | 1. 9 or 10 or 11 |
|  | 1. 4 and 8 and 12 |
| [quality indicators] | 1. ((quality adj3 improv$) or (quality adj3 enhance$)).ab,ti. |
|  | 1. 12 or 14 |
|  | 1. 4 and 8 and 15 |
|  | 1. Limit 16 to English language |
