# Supplementary material for: Measuring the quality of skin cancer management in primary care: A scoping review
Source: Australas J Dermatol. 2023 Mar 24;64(2):177–93. doi: 10.1111/ajd.14023 (PMC10952799; doi:10.1111/ajd.14023)
Supplement: Supplementary file 2 — Table S1. [file AJD-64-177-s001.docx]

**Table S1**. Groups of quality measures across all included studies

|  | **STRUCTURE** | | | | **PROCESS** | | | | | **OUTCOME** | | | |
| --- | --- | --- | --- | --- | --- | --- | --- | --- | --- | --- | --- | --- | --- |
|  | Diagnostic tools and equipment | Practitioner education and training | Diagnostic protocols and documentation | Treatment protocols and documentation | Prevention | Diagnostic process | Delays in care | Treatment process | Interpersonal process | Complications and adverse events | Patient-reported measures | Skin cancer recurrence | Long-term morbidity and mortality |
| Ahmadi (2017)^17^ |  |  |  |  |  | ✓ |  | ✓ |  |  |  |  |  |
| Aung (2019)^95^ |  |  |  |  |  |  |  | ✓ |  |  |  |  |  |
| Bibbins-Domingo (2016)^66^ |  |  | ✓ |  |  | ✓ |  | ✓ | ✓ |  |  |  |  |
| Blood (2021)^96^ |  |  |  |  |  |  |  |  | ✓ |  | ✓ |  |  |
| Botting (2016)^71^ |  |  |  | ✓ |  | ✓ | ✓ | ✓ |  | ✓ |  |  |  |
| Buckley (2013)^79^ |  |  |  |  |  | ✓ | ✓ | ✓ |  |  |  | ✓ | ✓ |
| Chuh (2020)^58^ | ✓ |  | ✓ | ✓ |  |  |  |  | ✓ |  |  |  |  |
| Cole (2018)^28^ |  |  |  |  |  |  |  | ✓ |  |  |  |  |  |
| Delaney (2012)^23^ |  |  |  |  |  | ✓ |  | ✓ |  |  |  |  |  |
| Dinnes (2018a)^60^ | ✓ | ✓ | ✓ |  |  | ✓ |  |  |  |  |  |  |  |
| Dinnes (2018b)^59^ | ✓ | ✓ | ✓ |  |  | ✓ |  |  |  |  |  |  |  |
| Doherty (2016)^80^ |  |  |  |  |  | ✓ |  | ✓ |  |  |  |  | ✓ |
| Gendreau (2017)^77^ |  |  |  |  |  | ✓ | ✓ |  |  |  |  |  |  |
| Guitera (2021)^78^ | ✓ |  |  |  |  | ✓ |  | ✓ |  |  |  | ✓ |  |
| Hajderevic (2014)^81^ |  |  |  |  |  | ✓ | ✓ | ✓ |  |  |  |  |  |
| Haw (2014)^82^ |  |  |  |  |  | ✓ |  | ✓ |  |  |  |  |  |
| Hay (2022)^83^ |  |  |  |  |  | ✓ |  | ✓ |  |  |  | ✓ | ✓ |
| Heppt (2020)^69^ |  |  | ✓ | ✓ | ✓ | ✓ |  | ✓ |  |  |  |  |  |
| Herschorn (2012)^61^ | ✓ | ✓ | ✓ |  |  | ✓ |  |  |  |  |  |  |  |
| Jimenez-Balcells (2021)^97^ |  |  |  |  |  |  |  |  |  |  |  |  | ✓ |
| Jobson (2022)^46^ |  |  |  |  |  | ✓ |  | ✓ |  |  |  |  |  |
| Kaiser (2014)^84^ |  |  |  |  |  | ✓ |  |  |  |  |  |  |  |
| Koelink (2014)^62^ | ✓ | ✓ | ✓ |  |  | ✓ |  | ✓ |  |  |  |  |  |
| Korgul (2018)^37^ |  |  |  |  |  |  |  | ✓ |  |  |  |  |  |
| Leiter (2020)^73^ |  |  |  | ✓ | ✓ |  |  | ✓ |  |  |  |  |  |
| Lott (2015)^89^ |  |  |  |  |  |  | ✓ |  |  |  |  |  |  |
| Maguire (2017)^92^ |  |  |  |  |  |  |  | ✓ |  |  |  | ✓ |  |
| Martinka (2016)^75^ |  |  |  |  |  | ✓ |  |  |  |  |  |  |  |
| Moreno-Ramirez (2016)^70^ |  |  | ✓ | ✓ |  | ✓ |  | ✓ |  |  |  |  |  |
| Moyer (2012)^74^ |  |  |  |  | ✓ |  |  |  | ✓ |  |  |  |  |
| Murchie (2013)^86^ |  |  |  |  |  | ✓ |  | ✓ |  | ✓ |  |  | ✓ |
| Murchie (2017)^85^ |  |  |  |  |  | ✓ |  | ✓ |  | ✓ |  |  | ✓ |
| Noels (2019)^67^ |  |  | ✓ |  |  |  |  | ✓ |  | ✓ |  | ✓ |  |
| Nolan (2021)^94^ |  |  |  |  |  |  |  | ✓ |  |  |  |  |  |
| Ramdas (2018)^93^ |  |  |  |  |  |  |  | ✓ |  |  |  |  |  |
| Renzi (2011)^90^ |  |  |  |  |  |  | ✓ |  |  |  |  |  |  |
| Smith (2014)^72^ |  |  |  | ✓ |  |  |  | ✓ |  | ✓ |  |  |  |
| Svensson (2020)^87^ |  |  |  |  |  | ✓ |  | ✓ |  |  |  |  |  |
| Van Rijsingen (2015)^76^ |  |  |  |  |  | ✓ |  | ✓ |  |  |  |  |  |
| Vestergaard (2020)^63^ | ✓ |  | ✓ |  |  | ✓ |  |  |  |  |  |  |  |
| Wakkee (2019)^34^ |  |  |  |  |  | ✓ |  | ✓ |  |  |  | ✓ |  |
| Walter (2012)^64^ | ✓ | ✓ | ✓ |  |  | ✓ |  |  | ✓ |  | ✓ |  |  |
| Wen (2020)^91^ |  |  |  |  |  |  | ✓ | ✓ |  | ✓ |  |  |  |
| Wernli (2016)^68^ |  |  | ✓ |  |  | ✓ |  | ✓ |  |  |  |  | ✓ |
| Wheatley (2018)^65^ |  | ✓ | ✓ |  |  |  |  |  |  |  |  |  |  |
| Wikstrom (2018)^88^ |  |  |  |  |  |  | ✓ | ✓ | ✓ |  | ✓ |  |  |
